# Prediction of Disease Progression, Treatment Response and Dropout in Chronic Obstructive Pulmonary Disease (COPD)

**DOI:** 10.1007/s11095-014-1490-4

**Published:** 2014-09-18

**Authors:** F. T. Musuamba, D. Teutonico, H. J. Maas, A. Facius, S. Yang, M. Danhof, O. Della Pasqua

**Affiliations:** 1 Gorlaeus Laboratories, Division of Pharmacology, Leiden Academic Centre for Drug Research, P.O. Box 9502, 2300 RA Leiden, The Netherlands; 2 Clinical Pharmacology Modelling & Simulation, GlaxoSmithKline, Uxbridge, UK; 3 Department of Pharmacometrics, Nycomed GmbH, Konstanz, Germany

**Keywords:** chronic obstructive pulmonary disease, disease modelling, disease progression, dropout, KPD model

## Abstract

**Purpose:**

Drug development in chronic obstructive pulmonary disease (COPD) has been characterised by unacceptably high failure rates. In addition to the poor sensitivity in forced expiratory volume in one second (FEV_1_), numerous causes are known to contribute to this phenomenon, which can be clustered into drug-, disease- and design-related factors. Here we present a model-based approach to describe disease progression, treatment response and dropout in clinical trials with COPD patients.

**Methods:**

Data from six phase II trials lasting up to 6 months were used. Disease progression (trough FEV_1_ measurements) was modelled by a time–varying function, whilst the treatment effect was described by an indirect response model. A time-to-event model was used for dropout

**Results:**

All relevant parameters were characterised with acceptable precision. Two parameters were necessary to model the dropout patterns, which was found to be partly linked to the treatment failure. Disease severity at baseline, previous use of corticosteroids, gender and height were significant covariates on disease baseline whereas disease severity and reversibility to salbutamol/salmeterol were significant covariates on E_max_ for salmeterol active arm.

**Conclusion:**

Incorporation of the various interacting factors into a single model will offer the basis for patient enrichment and improved dose rationale in COPD.

**Electronic supplementary material:**

The online version of this article (doi:10.1007/s11095-014-1490-4) contains supplementary material, which is available to authorized users.

## Introduction

Chronic obstructive pulmonary disease (COPD) is a chronic respiratory disorder that progresses slowly and is characterised by an obstructive ventilatory pattern, which is rarely reversible, very often related to tobacco smoking and which can lead to chronic respiratory failure (1). COPD is a major public health problem. Indeed, it is the fourth leading cause of morbidity and mortality in industrialised countries and is projected to rank fifth in 2020 in burden of disease caused worldwide by the World Health Organization (2,3). Even though exacerbations have been recognised as an important trait of the disease and is increasingly included in the evaluation of drug effects, forced expiratory volume in one second (FEV_1_) remains the most frequently used endpoint in clinical trials in COPD, with change from baseline (ΔFEV_1_) corrected for placebo being the primary measure of efficacy. In fact, FEV_1_ is currently considered to be one of the best predictors of patient survival and as such has been used as a prognostic marker for outcome (4). Consequently, regulatory authorities still recommend the use of measures of airflow obstruction improvement for registration trials of new bronchodilators.

From a therapeutic perspective, maintenance treatments are based on the use of bronchodilators which rapidly improve lung function. However, high failure rate is observed during the development of treatments for COPD. Numerous factors are known to contribute to this phenomenon, which can be clustered into drug-, disease- and design-related factors. Of particular relevance are the sensitivity of spirometric measures to patient demographic characteristics and the disease-related inclusion criteria such as gender, previous use of inhaled corticosteroids and disease severity at baseline. Yet, the majority of trials are usually conducted according to a fixed protocol, copied from one study to the next. Among the reasons for this conservatism are the high costs of these trials, which limit the willingness to experiment with designs, and concerns about potential failure because of deviations from traditional study designs. Another important point supporting current practice is the statistical evaluation of efficacy (*i.e.*, contrast between active agent and placebo) by changes from baseline at a single time point.

In other therapeutic areas in which similar concerns exist regarding the impact of confounding design factors, costs and time course of the response, the use of modelling and simulation has been advocated. In fact, the implementation of clinical trial simulations (CTS) allows evaluation of a single factor at a time. In addition, it provides the opportunity to investigate factors that have not been implemented yet and can therefore not be included in a meta-analysis. CTS enable statistical inferences from models and as such contrasts with the long-established calculations of statistical power, which are often based only on a point estimate of the variability of the clinical end point.

Here we show how a model-based approach can be used to discriminate drug effects from disease- and design-related factors on the overall treatment response. The aim of this investigation was therefore (1) to develop and validate a model for trough FEV_1_ including disease progression and dropout; and (2) to quantify the impact of relevant demographic and disease-related factors on treatment response. Our analysis is based on a kinetic-pharmacodynamic (KPD) model which was previously developed to overcome some of the limitations associated with lack of pharmacokinetic data. (5,6) This is a common problem during the development of inhaled compounds, for which plasma pharmacokinetics is not available and/or doses do not relate directly to treatment response. This concept has been successfully applied to model the effects of a novel long-acting bronchodilator in COPD in a recently published study (7).

Given the typical duration of efficacy trials in COPD, disease progression and dropout models were included to ensure unbiased estimation of treatment response. Dropout is considered to be informative (also called non random) when it is driven by the unobserved clinical response and non-informative otherwise (also called random and completely random dropout). In contrast to non-informative dropout that can be just described or predicted independently to the clinical response of interest, informative dropout must be jointly modelled with the response data in order to warrant reliable parameter estimates. Further characterisation of the patterns of dropout is therefore essential for models aimed at the prediction and simulation of response across a wide time span. Joint analysis of disease progression, treatment response and dropout is very often the most objective and reliable way to assess dropout patterns and account for its effects on parameter estimation. Joint models have been developed to describe the time course of biomarkers in long term diseases associated with relatively high dropout rates such as Parkinson’s disease (7,8), depression (9) and chronic viral infections (10). To date, joint modelling concepts have not been applied to FEV_1_ response in COPD.

## Methods

### Patients

The clinical data used for the analysis were extracted from Top Institute Pharma data repository. The data were anonymised and randomly combined prior to use. It consisted in a pool from 6 Phase II multicenter studies, containing placebo (*n* = 2273) and active treatment (salmeterol) (*n* = 270). All patients had a diagnosis of moderate to severe COPD as defined by the Global initiative for Chronic Obstructive Lung Disease (GOLD) guidelines (11). Moderate COPD implies that post-bronchodilator FEV_1_ values at the start of the study (T_0_) are between 50 and 79% of the predicted FEV_1_ for a subject with normal lung function, whereas for severe COPD post-bronchodilator FEV_1_ values range between 30 and 50% of the predicted FEV_1_. Very severe patients (post-bronchodilator FEV_1_ at T_0_ < 30%) were excluded from the trials. Spyrometry (trough FEV_1_) was performed after administration of bronchodilators at intervals ranging between two and a maximum of four weeks by trained clinical staff. The study drugs were inhaled salmeterol (50 μg) or matched placebo administered as a twice daily regimen. Patients were not allowed to take another bronchodilator during the course of treatment, other than rescue medication (*e.g.*, in case of exacerbations). Table I summarises patient demographics and disease related factors at the start of the trials, including study duration and number of patients enrolled. The protocols were approved by the local institutional review boards, and all patients agreed to sign an informed consent form. We have used data from patients enrolled into three of the six studies for model building purposes and included those in the remaining studies during the model validation procedures. The final model parameter estimates refer therefore to the total pool of patients in all six studies.

**Table I:** COPD Patient Demographics and Study Characteristics

	Median [range]
Number of patients (studies 1–6)	2543
Reversibility (mL)	56
Age (years)	65 [40–90]
Sex (% of females)	30
Height (cm)	170 [135–203]
Patients with severe COPD (%)	67
Patients with reversible COPD (%)	26
Patients with a previous use of inhaled corticosteroids (PICS) (%)	40
Smoking status (% of smokers)	42
Dropout (% of dropout)	13
Study duration (weeks)	167

### Modelling and Covariate Analysis

Nonlinear mixed effects modelling was performed using NONMEM v7.1.2 (double precision, Icon Development Solutions, Ellicott City, MD, USA) and Perl-speaks-NONMEM (PsN)-toolkit, (12) a programming library containing a collection of computer intensive statistical methods for non-linear mixed effects modelling, Xpose 4.0 (13) and R (14). First order conditional estimation method with interaction (FOCEI) was first used to model disease progression and drug effects. Subsequently, dropout patterns were analysed using the Laplace estimation method, which is deemed to be more appropriate to jointly model continuous and categorical data in NONMEM. The models for disease progression, drug effects and dropout were developed simultaneously. For the sake of completeness, the disease and drug effect parameter estimates obtained with and without dropout were compared to assess the influence of informative dropout.

#### Base Model

##### Disease and Drug Effects Model

The process describing FEV_1_ changes over time was modelled using an indirect response model, as described by the following differential equation

1 $$ \frac{dFE{V}_1}{dt}= Kin- Kout\times FE{V}_1 $$

where the change in the observed FEV_1_ over time (*d*FEV_1_/*d*t) is controlled by a zero-order process parameterised as a synthesis rate constant (Kin) and first order elimination process (Kout). The disease status at baseline was considered to be described by the ratio between Kin and Kout. The disease progression was modelled as a linear decline in the baseline status as follows:

2 $$ Dis= Int\_ Dis- Slope\_ Dis\times Time $$

with

3 $$ Dis=\frac{Kin}{Kout} $$

where Dis is the disease status, Int_Dis is the disease status at the start of the clinical trial, and Slope_Dis is the daily decline of Dis due to disease progression.

The effect of bronchodilators was modelled using a nonlinear Emax function, in which the maximum effect is proportional to an apparent potency parameter (EDK_50_). Four different models were tested with drug effects incorporated either additively or multiplicatively on Kin or on Kout. Examples are provided below for incorporation on Kin:

4 $$ Kin= TVKin+\left(\frac{A\times Emax}{ED{K}_{50}+A}\right) $$

5 $$ Kin= TVKin\times \left( 1+\frac{A\times Emax}{ED{K}_{50}+A}\right) $$

where TVKin is the typical value of Kin in the absence of drug effects, EDK_50_ is the drug exposure associated with half of the maximum effect, Emax is the maximum drug effect, and A is the drug exposure marker. Different parameterisations were tested for characterisation of the exposure markers including:

- d(A)/dt = −KDE×A and A_0_ = Dose
- A = Dose
- A = D×KDE with d(D)/dt = −KDE×D and D_0_ = Dose

where A is the amount (a), the dose (b), or the input rate (c) of drug into the effect compartment, D is the dose at time 0 KDE is the first order elimination rate constant for the bronchodilator and A_0_/D_0_ are the initial values for A/D.

Given that all the patients belonging to the active arms of the different trials were taking the same dose of bronchodilators, a prior function ($PRIOR) was used in NONMEM to stabilise the estimation of correlated parameters (*i.e.*, Emax and EDK_50_). In addition, the ratio between Emax and EDK_50_ was first estimated and EDK_50_ was computed subsequently. Prior information on the exposure response relationship was derived from a model using data from a previously published study (15). Emax and EDK_50_ distributions were directly taken from the NONMEM output file of the previous model. The parameter estimates for Emax and EDK_50_ were 0.78 and 4 μg, respectively. The values used as priors on interindividual variability (OMEGA) were 0.24 and 0.14 with a covariance term of 0.1. The NONMEM variance/covariance matrix was used to weight the prior information. The degrees of freedom for the OMEGAs were based on the number of patients included in the historical study and set to 24.

##### Dropout model

Dropout was modelled using a time-to-event model (10,16,17). The probability of a having a dropout at any given time was predicted by describing the hazard (h(t)) associated with the dropout (*i.e.*, event). Different hazard models were tested including constant, exponential and Weibull models, as previously described in (16,17).

As time passes the cumulative hazard predicts the risk of having the event over the interval 0-t. The risk (cumulative hazard) was obtained by integrating hazard with respect to time:

6 $$ Cumulative\kern0.5em  hazard={\displaystyle {\int}_0^th(t)} $$

On the other hand, the probability of remaining in the trial (*i.e.*, of not dropping out) was predicted by a survivor function from the cumulative hazard.

7 $$ Survivor(t)=P\left(T>t\right)= \exp \left(-{\displaystyle {\int}_0^th(t)}\right) $$

The probability of dropping out at any given time was predicted by the probability density function (pdf(t)). The pdf was calculated from the survivor function and from the hazard at the specific time as follows:

8 $$ pdf(t)= Survivor(t)\times h(t) $$

The probability of dropping out within an interval was modelled by taking the difference between the survivor at the first time after dropout and the survivor at last observed time before dropout.

Dropout patterns were characterised using likelihood ratio tests by including additional parameters on the initial hazard term. For example when a constant hazard model was tested, the following equation was used:

9 $$ h(t)={\beta}_0\times {e}^{\beta_1\times FE{V}_1+{\beta}_2\times IPRED} $$

where IPRED represents the individual predicted FEV_1_ at time (t), FEV_1_ is the observed measure at available sampling times, the coefficient β_0_ and the exponents β_1_ and β_2_ describe missingness completely at random, at random and non- random, respectively.

##### Inter-Individual and Unexplained Variability Model

Exponential models were used for inter-individual variability (IIV) on model parameters. The value of a parameter in the i^th^ individual (P_ij_) was a function of the typical value of the parameter (TVP*)* and of the individual deviation initially represented by η_i_, which describes the inter-patient variability term for the i^th^ patient. The ηs in the population were assumed to be normally distributed random variables with zero mean and a variance that is estimated as part of the model:

10 $$ {\mathrm{P}}_{\mathrm{ij}}=\mathrm{TVP}\times \exp \left(\upeta \mathrm{i}\right) $$

The covariance between different parameters was also assessed using additional random effects terms using the OMEGA BLOCK() option in NONMEM. η terms were only maintained in the model when they improved the fitting as assessed by comparing the NONMEM objective function values with and without the inclusion of the random effects. A difference of at least 3.84 points was necessary to keep the random effects term in the model.

Additive, proportional and mixed error models were tested for the residual error as shown in Eqs. 11–13:

11 $$ \mathrm{Y}=\mathrm{IPRED}+{\upvarepsilon}_{\mathrm{add}} $$

12 $$ \mathrm{Y}=\mathrm{IPRED}\times \left(1+{\upvarepsilon}_{\mathrm{prop}}\right) $$

13 $$ \mathrm{Y}=\mathrm{IPRED}\times \left(1+{\upvarepsilon}_{\mathrm{prop}}\right)+{\upvarepsilon}_{\mathrm{add}} $$

where Y and IPRED represent the observed and individual predicted FEV_1_ values, respectively. ε_add_ and ε_prop_ are the additive and the proportional error terms on FEV_1_ values, respectively. Epsilon was supposed to be normally distributed, zero-mean random variables with variance (σ^2^) terms that are estimated as part of the population model-fitting process.

##### Model Selection

Structural model selection was based on 1) the change in the minimum objective function value (MOFV), with a decrease of at least 3.84 points being required for additional parameter inclusion, 2) the plausibility and the precision of parameter estimates and 3) the qualitative evaluation of goodness-of-fit plots. The precision of parameter estimates, expressed as standard error of estimates, was generated by the covariance option in NONMEM. Goodness-of-fit plots comprised predicted and individually predicted *versus* observed data, conditional weighted residuals (18) (CWRES) *versus* time after dose, as well as the normalised prediction distribution errors (NPDE) (19). The precision of parameter estimates, expressed as standard error of estimates, was generated by the covariance option in NONMEM.

#### Covariate Model

A stepwise inclusion/exclusion approach was used to investigate the relationship between different structural model parameters and demographic factors. To explain inter-patient variability on disease and on drug-related parameters, and to reduce the unexplained residual variability, relationships were investigated for age, sex, height, BMI, weight, severity, reversibility, previous use of inhaled corticosteroids (PICS). Individual empirical Bayes estimates of parameters were generated and their correlation with each covariate being evaluated separately during the exploratory steps of the covariate analysis. The log-likelihood ratio is assumed to be *χ* ^2^-distributed. A difference in objective function value of 3.84 is considered to be significant at *p* < 0.05 with one degree of freedom (difference of one parameter between the two nested models). Continuous variables (age, weight, height) were centred to their median values and tested on the parameters using a power model (see Eq. 14). Categorical variables were entered in the model in a multiplicative manner (see Eq. 15). This is exemplified below for height and gender:

14 $$ Int\_ Dis= TVInt\_ Dis\times {\left(\raisebox{1ex}{$ HT$}\!\left/ \!\raisebox{-1ex}{$ HT med$}\right.\right)}^{\uptheta_{cov}} $$

15 $$ Int\_ Dis= TVInt\_ Dis\times {\uptheta}_{cov}\left[ in\  case\  of\  males\right] $$

where Int_Dis is the disease baseline status (*i.e.*, baseline FEV_1_ at *t* = 0 expressed in L), TVInt_Dis is the typical value for disease baseline intercept (in case of continuous covariate, TVInt will be the disease baseline status for a patient with median covariate (here height) value and for categorical covariates, it will be the value for the reference category (here females)), Θ_COV_ is the relative change in baseline intercept due to covariate effect. HT is the patient body height and HTmed is the median body height in the dataset.

A backward deletion process was used to complete the covariate model building. Only covariates which upon deletion caused an increase in MOFV > 11 points (*χ* ^2^ *p*-value ≤ 0.001) were retained in the final model.

#### Final Model Evaluation

An initial assessment of the predictive performance of the model was carried out by using the parameter estimates from the model building studies 1–3 (*n* = 1154 patients) to describe the trough FEV_1_ response profile in the remaining studies 4–6 (*n* = 1389 patients). Bootstrapping and simulations were used to internally validate the final model. 100 bootstraps were generated using PsN and confidence intervals were obtained for all model parameters. NPDE-related diagnostic plots (19) and mirror plots were then generated to assess overall model performance.

Finally, visual predictive checks (VPC) were performed taking dropout into account. Observed and simulated FEV_1_ values *vs.* time were presented in conjunction with Kaplan Meier plots to assess the suitability of the dropout model.

## Results

**Fig. 1:**
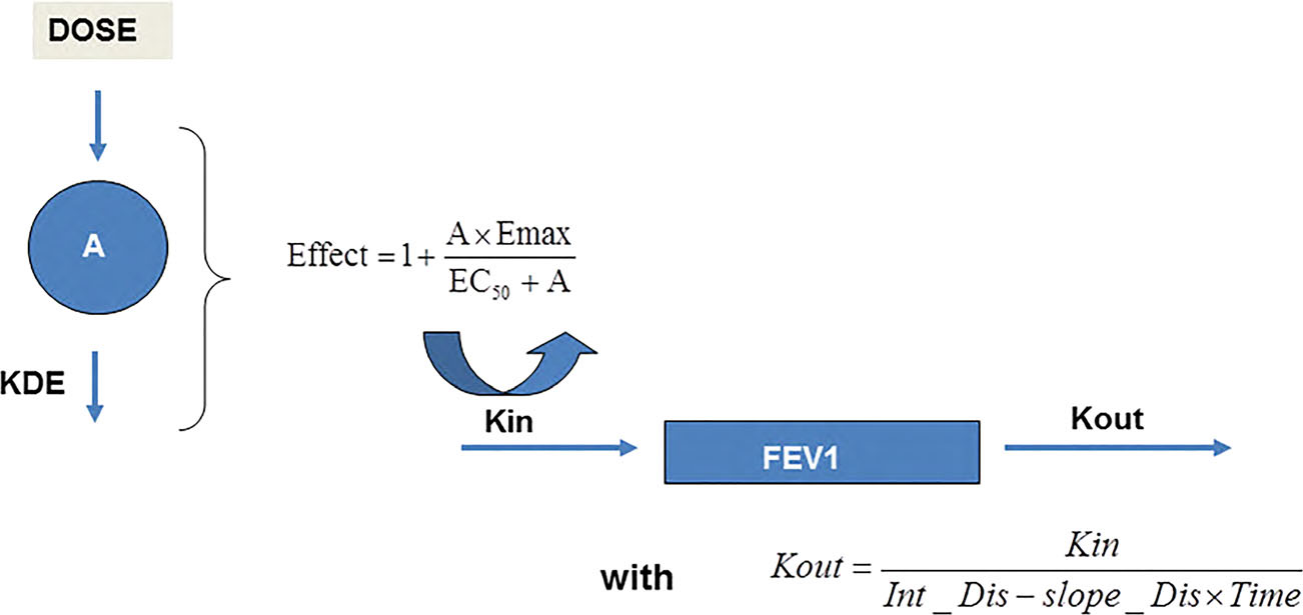
Schematic representation of the structural model. Bronchodilatory effect on FEV_1_ is described by an Emax function using a KPD model as input for drug exposure. The overall response to treatment accounts for the course of disease, *i.e.*, the natural decrease in FEV_1_ over time, which has been parameterised in terms of an indirect response model. See text for further explanation of the parameters.

Disease progression (trough FEV_1_ measurements) was modelled by a time–varying function. The treatment effect was described by an indirect response model using an Emax model in a multiplicative manner on Kin. In addition, a time-to-event model with constant hazard was used for dropout. A schematic representation of the structural model for disease progression and drug effects is shown in Fig. 1. The following disease progression and drug-related parameters were estimated: disease baseline intercept (Int_Dis), disease baseline slope (Slope_Dis), zero order input rate constant for the biological response (Kin), first order elimination rate constant (KDE), maximum drug effect (Emax) and the ratio between the apparent potency (EDK_50_) and Emax. Two parameters (β_0_ and β_2_) were necessary to model the dropout patterns. A constant hazard (β_0_) of 0.06 was estimated, which was increased in case of treatment failure (*i.e.*, lower FEV_1_ values). The inclusion of β_2_ in the model led to a decrease of 15 points in the MOFV as compared to the model only including β_0._ The best fit was obtained with interindividual variability included on the disease slope, the disease baseline intercept, Emax, KDE, EDK_50_ and Kin. All covariance terms turned to be lower than 0.1 and their removal did not appear to decrease model performance or goodness of fit. Random effects were not imputed on dropout parameters. Residual errors were best described by an additive model. The parameter estimates for the base model are summarised in Table II.

**Table II:** Overview of Base and Final Model Parameter Estimates

Parameter	Base model [RSE]^a^	Final model [Bootstrap 90% CI]
Θ_TVEmax_	0.34 [0.04;11.5]	0.36 [0.05;0.81]
Θ_EDK50/Emax_^b^ (μg)	10.33 [0.64;20.02]	8.66 [5.19;11.39]
Θ_KDE_ (hr^−1^)	54.46 [1.64;1806]	52.46 [12.18;65.14]
Θ_Int_Dis_ (disease intercept) (L)	0.9 [0.3;2.99]	1.16 [1.06;1.19]
Θ _Slope_Dis_ (disease slope) (L/day)	−0.82 [−1.93;-0.02]	−0.596 [−0.214;]
Θ_Kin_ (L/day)	0.04 [0.001;1.32]	0.0004 [0.0001;0.0032]
Θ_severity on Emax_	–	0.94 [0.08;0.99]
Θ_reversibility on Emax_	–	1.05 [1.02;1.52]
Θ_severity on Int_Dis_	–	0.62 [0.22;0.84]
Θ_sex on Int_Dis_	–	0.82 [0.13;0.97]
Θ_PICS on Int_Dis_	–	0.92 [0.77;0.94]
Θ_height on Int_Dis_	–	1.90 [1.48;2.52]
Initial hazard term (β_0_)	0.006 [0.0001;0.33]	0.006 [0.000;0.041]
Proportional hazard term for informative dropout (β_2_)	−0.843 [−0.11;−1.57]	−0.880 [−0.19;−0.92]
IIV_EDK50_ (CV%)	8 [0.01;15.9]	–
IIV_Emax_ (CV%)	76 [0;153]	70 [32;108]
IIV_Int_Dis_ (CV%)	35 [0;81]	25 [12;63]
IIV_Slope_Dis_ (CV%)	16 [0;33]	–
IIV_Kin_ (CV%)	10 [3;19.7]	8 [11;28]
σ (L)	0.36 [0.021;0.39]	0.13 [0.08;0.24]

^a^ *RSE* relative standard errors; values computed from standard error of estimates generated by covariance step in NONMEM

^b^EDK_50_ estimates were based on a parameterisation relative to Emax

**Fig. 2:**
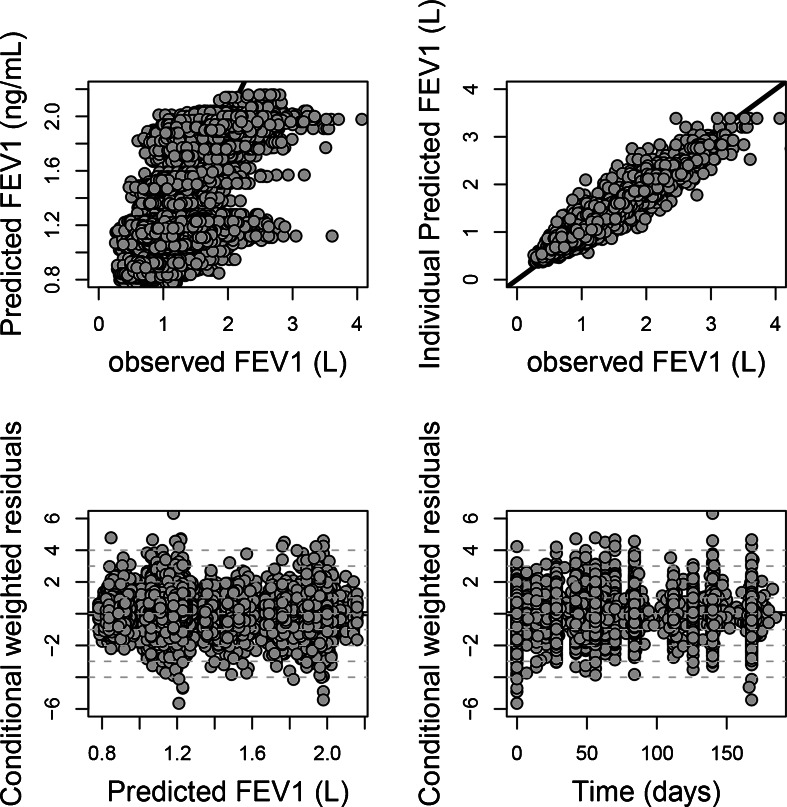
Basic goodness-of-fit plots for the final model, including population and individual predicted *vs.* observed FEV_1_, conditional weighted residuals *vs.* time and individual predicted FEV_1._

During covariate model building, disease severity at baseline, previous use of inhaled corticosteroids, gender and height were retained as significant covariates on Int_Dis whereas reversibility to salbutamol/salmeterol and disease severity at baseline were significant covariates on Emax for the active arm (salmeterol). All covariates were included in the final model in a as shown in Eqs. 14 and 15. A summary of the final parameter estimates including the aforementioned covariates is provided in Table II. All model parameters were well estimated, as shown by the relative low standard error of estimates (<50%) and by shrinkage levels <30% for all random effects parameter values. Basic goodness-of-fit plots also showed good model predictive performance (see Fig. 2).

**Fig. 3:**
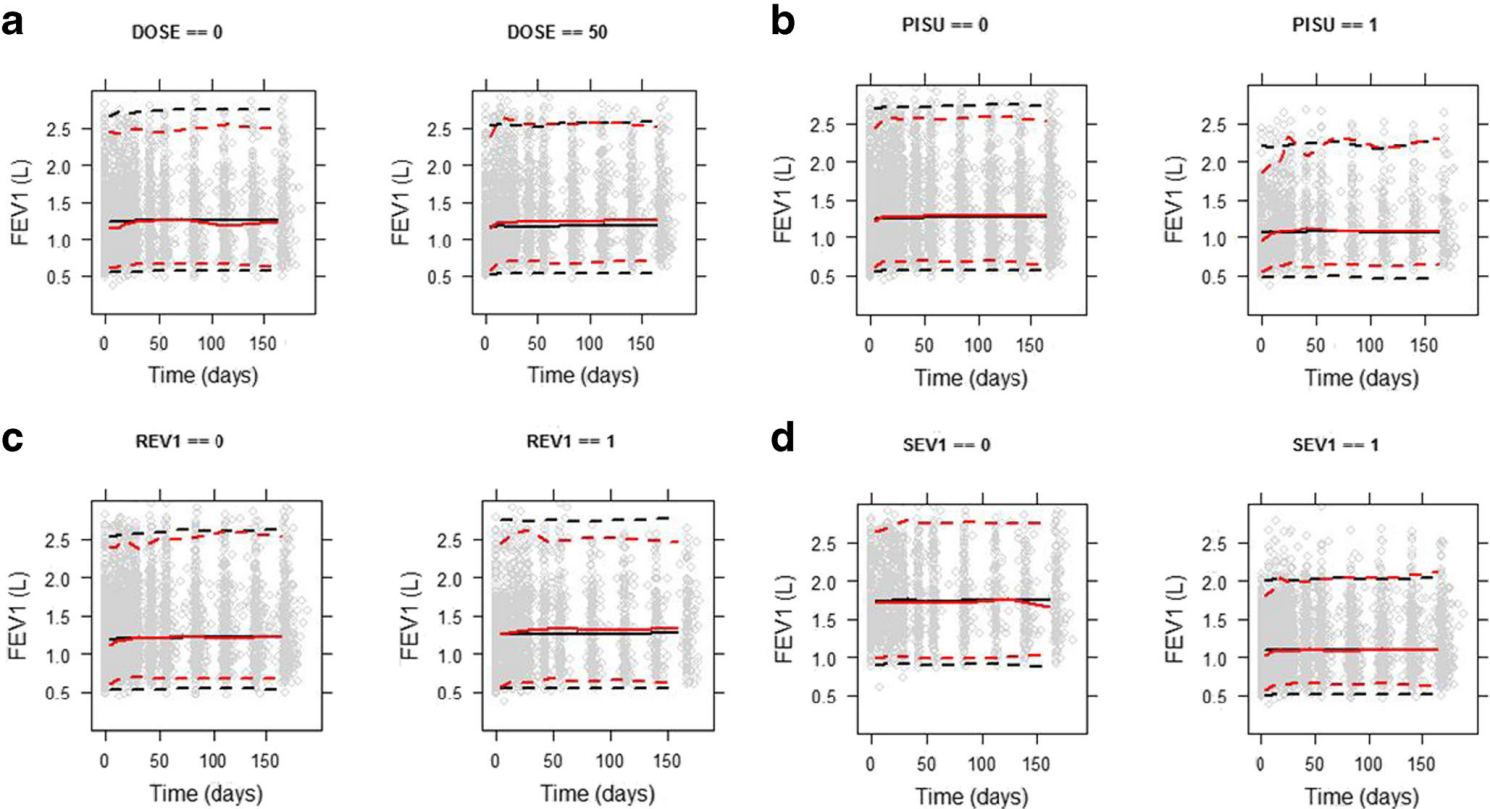
Visual predictive check stratified by (**a**) treatment (*left panel*: placebo, *right panel*: 50 μg dose), (**b**) by previous use of inhaled corticosteroids [PISU] (*left panel*: no, *right panel*: yes), (**c**) by reversibility to salbutamol/salmeterol [REV] (*left panel*: non-reversible, *right panel*: reversible), and (**d**) by severity status [SEV] (*left panel*: non-severe, *right panel* severe). *Grey dots*: observed concentrations, *black dotted lines*: limits of the 95% prediction intervals for the observations, *black dotted lines*: limits of the 95% prediction intervals for the simulations, *black continuous line*: median line for the observations, *red continuous line*: median line for the simulations. See text for further details on the scenarios selected for the VPC.

**Fig. 4:**
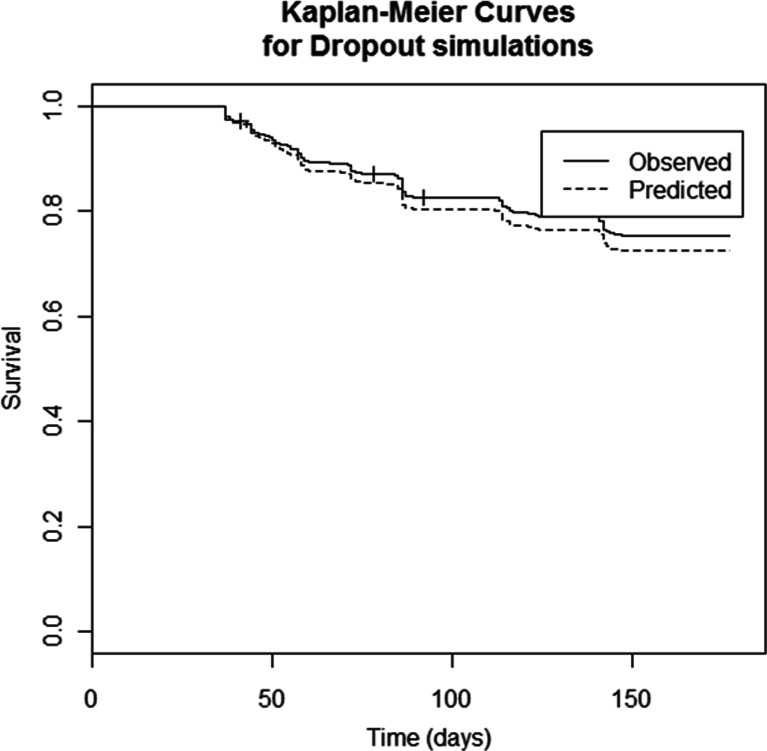
Kaplan Meier plots of observed (*black*) and simulated (*grey*) dropout for a subset of the pooled studies used for the current analysis.

Validation of the final model by external validation procedures and bootstrapping yielded good predictive performance. All observed parameter values obtained by bootstrapping were found to be within the 90%-confidence interval (*n* = 100 bootstraps). In addition, as shown by the visual predictive check in Fig. 3, most of the observed data in the validation subset of data were distributed within the 5th and 95th percentiles of the prediction intervals. For the sake of clarity, similar predictive performance is observed when the response profiles were split by severity status (severity status being the covariate with the highest effect size) and by dose group. The overlap between predicted and original distributions shows that the model accurately captures drug, disease and dropout effects (Fig. 4).

**Fig. 5:**
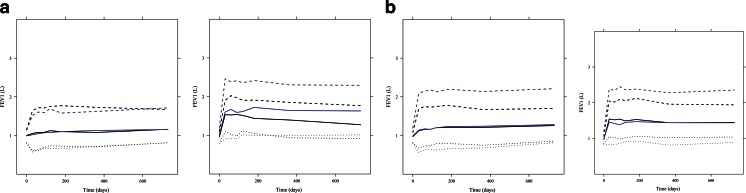
Simulations (*n* = 1000) of four scenarios based on the final model. In *blue*: median and 95% prediction intervals for the placebo and in *black*: median and 95% prediction intervals for the active arm. (**a**) *left panel*: non-reversible and severe patients, right panel: reversible and non-severe patients; (**b**) patients with height >172 cm and previous use of inhaled corticosteroids, *right panel*: patients with height >172 cm and no previous use of inhaled corticosteroids. To ensure appropriate comparisons between the groups, the remaining covariates were kept in a balanced manner in each group.

One of the interesting findings from our analysis is that the change in FEV_1_ values over time was found to be dependent on the baseline characteristics. For instance, as shown in Fig. 5, patients with a more severe disease status and unfavourable covariate characteristics at baseline will have a slower evolution over time and will be less sensitive to drug effects (see Fig. 5(a) and (b)). The correlation between change in FEV_1_ values over time and baseline characteristics is consistent with indirect drug effects, as described by increasing zero-order processes. This is particularly relevant for salmeterol, given that baseline severity status shows a higher effect on the disease slope than the active treatment itself when salmeterol is administered at 50 μg doses according to a twice daily-dosing regimen. On the other hand, higher sensitivity to the drug effects was more pronounced in patients who show reversibility to salbutamol/salmeterol at baseline.

## Discussion

We have shown the performance of a joint model for trough FEV_1_ in a large COPD patient population, which takes into account disease progression, drug effects and dropout. Despite the wide variation in the individual time course profiles, our approach resulted in a model with appropriate predictive performance, as shown by the precision in parameter estimates and the quality of goodness of fit for both treated and untreated arms of the study population.

Albeit not explicitly mechanistic, this model is consistent the known underlying processes associated with the pathophysiology of COPD. In fact, a more descriptive parameterisation of the underlying disease processes may not be feasible with data from typical Phase II clinical trials. Moreover, previous research has shown that no clear correlation exists between biomarkers of the underlying inflammatory condition and common spirometric measures, such as trough FEV_1_ or serial FEV_1_, making it difficult to define a fully mechanistic parameterisation for disease processes (20,21). Treatment response on FEV_1_ was therefore best described by an Emax model.

Parameterisation of the disease processes was based on the assumption that spirometric measures result from a series of putative turnover (*i.e.*, synthesis and degradation) processes, which can be modelled as a zero-order input rate and leading to FEV_1_ increase and a first-order elimination rate leading to FEV_1_ decrease. The synthesis-related processes include all the factors that can enhance the respiratory capacity, such as the ciliary function, the physiological beta-adrenergic tonus and the drug-related effects (*i.e.*, beta agonism and muscarinic antagonism) whereas the degradation-related processes comprise the secretory (mucus production) function of the epithelial cells and inflammatory components which restrict airway reversibility in COPD. This type of parameterisation has been used to describe several physiological responses involving inflammatory conditions (22–24). As indicated previously, our modelling approach incorporated disease progression, which is expressed as a time-varying increase in the ratio between degradation and synthesis of response. This model is therefore consistent with the results of several previously reported trials (26–30) showing that bronchodilators effects (including β_2_-agonists, such as salmeterol and M_2_ antagonists, such as tiotropium) in COPD are characterised by a rapid improvement of the lung function followed by a long steady phase. In addition, our analysis replicates the unexplained variability observed in FEV_1_ in previously published models, which was estimated to be approximately 0.1 L. The additive residual variability in FEV_1_ was 0.13 L in our model.

The different covariates included in our final model were measured or recorded at the start of the studies (disease severity, gender, previous use of inhaled corticosteroids, body height). Whilst their role in the long-term prognosis of COPD has been previously demonstrated (31,32), this is the first time that their influence on FEV_1_ is described in a quantitative manner using a joint model, in which dropout and disease progression are considered concurrently. In addition, our choice of parameterisation has enabled the discrimination between disease and drug-related changes in response. This approach also shows how dropout affects drug-specific parameters. Indeed, when fitting the model to the data without taking dropout into account, differences of 37 and 55% where found in the obtained estimates of EDK_50_ and Emax, respectively.

Today’s pursuit for disease-modifying drugs and personalised therapies requires one to understand the determinants of response and variability as the basis for subsequent evaluation of candidate molecules and tailored dosing regimens. Equally important is our understanding of the role of such factors on trial design during the evaluation of safety and efficacy (33). Unfortunately, for many clinical experts modelling results do not provide immediate evidence of the impact that such a parametric representation of disease progression can have on the development of novel anti-inflammatory drugs for COPD. From a theoretical perspective, the availability of drug- and system-specific parameters enables us to predict the outcome of novel interventions and handle potential confounders of response as covariates. Furthermore, our analysis clearly shows that despite the controversy regarding the use of FEV_1_ as a marker of disease progression (34), further insight into the underlying disease processes can provide the basis for patient selection and enrichment in clinical trials.

As previously stated, the primary purpose of this model will be its use in clinical trial simulations. The added value of scenario analysis and quantitative assessment of the impact of enrichment procedures on treatment response in Phase IIa and IIb trials will be the object of separate report in which a series of trial designs and treatment options are scrutinised. Yet, from a purely statistical perspective, two immediate lessons can be learnt from this analysis. First, the inclusion of a dropout model has highlighted the implication of non-random data missingness in longitudinal efficacy and safety trials. Of particular interest is the underestimation of the disease status (described by FEV_1_ values) and consequently inaccurate estimation of the drug effects. Second, the potential bias in effect size when comparing changes in FEV_1_ relative to baseline (ΔFEV_1_) for different treatment arms (active arm *vs.* placebo) based on traditional hypothesis testing (*e.g.*, unpaired ANOVA test). This approach can easily fail to identify drug effects when confounding effects such as baseline characteristics are not explicitly incorporated as covariates.

We need to acknowledge a few limitations in the analysis described here. It must be noticed that parameter identifiability issues arise when sampling intervals are wide and data on different treatment levels do not exist (*i.e.*, only one active dose level is usually evaluated in a clinical protocol). This issue results in parameter uncertainty, as shown by the wide confidence intervals obtained for most parameter estimates. This limitation is unlikely to be overcome unless phase II protocols are designed with the explicit objective of characterising exposure-response relationships (33). In addition, estimation of drug-specific parameters such as Emax and EC_50_ could not be obtained solely from the available clinical data. The use of prior information was required under the assumption of comparable, exchangeable experimental conditions across studies. We also emphasise the role of exclusion criteria on the identification of covariates and subsequent estimation of covariate effect size. Prediction of treatment effect in a real-life population requires one to account for those patients who are normally excluded from clinical trial protocols.

## Conclusion

Using a joint model, we have shown that gender, height, disease severity at baseline, previous use of corticosteroids and dropout rates should be taken into consideration when designing early trials. We anticipate its value in the design of prospective trials for which currently used statistical approaches are inadequate or incomplete. Incorporation of the various interacting factors into a single model will offer the basis for patient enrichment and improved dose rationale in COPD.

## Electronic supplementary material

Below is the link to the electronic supplementary material.

Figure 1S (DOCX 473 kb)

## Abbreviations

BMI: Body mass index
COPD: Chronic obstructive pulmonary disease
CTS: Clinical trial simulations
EDK_50_: Apparent potency parameter corresponding to the exposure associated with half of the maximum effect
Emax: Maximum effect
FEV_1_: Forced expiratory volume in one second
FOCEI: First order conditional estimation method with interaction
GOLD: Global Initiative for Chronic Obstructive Lung Disease
Int_Dis: The disease status at baseline, *i.e.*, at the start of the clinical trial
Kin: Zero-order process parameterised as a synthesis rate constant
Kout: First-order elimination constant
KPD: Kinetic-pharmacodynamic
MOFV: Minimum value of objective function
PICS: Previous use of corticosteroids
PsN: Perl-speaks-NONMEM
Slope_Dis: Parameter describing the daily decline of FEV_1_ due to disease progression
VPC: Visual predictive check
