# Supplementary material for: Prediction of Disease Progression, Treatment Response and Dropout in Chronic Obstructive Pulmonary Disease (COPD)
Source: Pharm Res. 2014 Sep 18;32(2):617–27. doi: 10.1007/s11095-014-1490-4 (PMC4300418; doi:10.1007/s11095-014-1490-4)
Supplement: Supplementary file 1 — (DOCX 473 kb) [file 11095_2014_1490_MOESM1_ESM.docx]

SUPPLEMENTARY MATERIAL

(A) (B)

Figure 1S: Diagnostics for the predictive performance in subsequent applications of the model in clinical trial simulations. (a) normalised predictions distribution errors (NPDE), (b) mirror plots for the placebo arm of study 1.
